# Newly developed CeO_2_ and Gd_2_O_3_-reinforced borosilicate glasses from municipal waste ash and their optical, structural, and gamma-ray shielding properties

**DOI:** 10.1038/s41598-024-63207-4

**Published:** 2024-06-13

**Authors:** E. M. Abou Hussein, S. E. Shaban, Y. S. Rammah, M. Hamed Misbah, M. A. Marzouk

**Affiliations:** 1 Radiation Chemistry Department, National Center for Radiation Research and Technology (NCRRT), Egyptian Atomic Energy Authority (EAEA), Cairo, Egypt; 2 Safeguards and Physical Protection Department, Nuclear and Radiological Safety Research Center (NRSRC), Egyptian Atomic Energy Authority (EAEA), Cairo, Egypt; 3 Physics Department, Faculty of Science, Menoufia University, Shebin El Koom, 32511 Egypt; 4 Institute of Nanoscience and Nanotechnology, Kafrelsheikh University, Kafrelsheikh, 33516 Egypt; 5 Glass Research Department, National Research Centre, 33 El Bohouth St. (Former El Tahrir St.), Dokki, P.O. 12622, Giza, Egypt

**Keywords:** Municipal waste, Borosilicate glasses, Rare earth ions, Photoluminescence, Radiation shielding parameters, Environmental social sciences, Chemistry, Materials science

## Abstract

From the useless municipal solid waste (MSW) ashes, CeO_2_, Gd_2_O_3_ and CeO_2_ + Gd_2_O_3_ doped borosilicate glasses were organized via melting-quenching procedure. Various optical, structural, physical and radiation shielding parameters were examined towards the influence of 100 kGy of γ-radiation. UV–visible NIR spectra revealed UV peaks at 351, 348 and 370 nm corresponding to the trivalent states of Ce^3+^ and Gd^3+^ ions, while, photoluminescence (PL) spectra displayed asymmetric broad excitations of Ce^3+^ and Gd^3+^ ions due to 4f → 5d transitions, and emission intense bands at 412, 434, and 417 nm. CIE chromaticity shows that Gd^3+^ ions increase the luminescence of Ce^3+^. FTIR absorption bands revealed an overlapping between tetrahedral groups of silicate (SiO_4_), with trigonal (BO_3_) and tetrahedral (BO_4_) units of borate. The influence of 100 kGy obtains quite reduction in UV–visible NIR and PL peaks, large stability in FTIR and ESR spectra, and stability of thermal expansion coefficient (CTE) as well. The whole data revealed optical, structural and physical stability of glasses after irradiation besides an enhancement in microhardness owing to more structural compactness and high bonding connectivity. Radiation shielding parameters from Phy_-_X/PSD program showed higher values of mass (MAC) and linear attenuation coefficients (LAC), and effective atomic number (Z_eff_) in the order of; glass _Ce+Gd_ > glass _Ce_ > glass _Gd_. Ce + Gd doped glass revealed also the lowest half value layer (HVL) comparing to other shielding commercial concretes. The study recommends the beneficial and economical use of the useless MSW ash to produce CeO_2_ and/or Gd_2_O_3_ borosilicate glasses with hopeful radiation shielding features.

## Introduction

Due to the modern rapid technological development, highly energetic radiation is used in various life fields such as agriculture, industry, medicine, space and energy^1^. To save humans and all environments from different radiation dangers, presence of radiation shielding materials becomes an urgent need. Some factors like time of exposure to radiation source, distance between the source and worker, and the type of shielding material, control all the material shielding efficacy. However, concrete is deliberated as the simplest and the most economical shield, its usage area is reasonably restricted because of many disadvantages obtained from the prolonged exposure to nuclear radiation such as shrinkage, settlement, weather and load, thermal stress, hydration heat plus its nature as opaque material^2^. Therefore, request of transparent radiation shielding materials is highly required. Glass can achieve this target and recently specific glass compositions are broadly used in radiation shielding purposes attributable to their value in protecting radiation workers, specialists and patients from natural and artificial radiation risks^3^. Besides the flexible structure of glass and the ease of hosting different metal oxides into glass structure, non-toxicity, chemical stability and optical transparency are also available in many types of glass and highly required e.g., borosilicate glasses.

Most industrial glassy materials are made up of borosilicate glasses such as laboratory equipment’s (test tubes, flasks, beakers, thermometers, Erlenmeyer …) and many other life applications. Recently borosilicate glasses have imperative applicable role in radiation fields, as they can be used widely in immobilization or disposal of radioactive wastes as well as radiation shielding and dosimeter glasses. Borosilicate glasses have a large capability for transmitting visible light and have lower transition temperature than SiO_2_ glasses. They are also resilient to acidic/ basic leaching solutions and organic materials, in addition to their resistance to high temperature changes, they are so called as “thermal glass”^1,4^.

The municipal solid waste (MSW) represents a giant problem in highly populated countries, as large tons amounts of residual ashes are left behind after sorting the recyclable wastes. Accumulation of these ashes represents another problem due to the resulting pollution of air, soil, ground water and the whole environment, besides the bad appearance of landfillings. Accordingly, using theses residual ashes to produce an applicable industrial product is a novel interesting approach^5,6^. The residual ash contains various metals related to the different components of wastes. This variety help in preparing richly glass compositions from borosilicate glasses that have precise properties over silicate and borate glasses such as high thermal resistance and chemical durability, mechanical reliability and specific optical properties^5–7^, associating to their complex structure of both SiO_2_ and B_2_O_3_ networks.

All over the periodic table elements, lanthanides or rare earth (RE) ions (such as La, Ce, Pr, Nd, Pm, Sm, Eu, Gd, Tb, Dy, Ho, Er, Tm, Yb, and Lu) have superior properties correlated to their high coordination number and large atomic sizes^8^, so they acquire their materials many commercial applications in lasers emitting (visible light or near-infrared radiation), lighting, telecommunications, white light emitting diodes (tricolor W-LEDs), photovoltaic and optoelectronic devices^9,10^, and recently for radiation shielding and dosimetry applications. RE ions are described by the electronic configuration; [Xe] 4*f*^12^.6*s*^2^ with outer-shell configuration; 5*s*^2^5*p*^6^6*s*^2^, the trivalent state acts as the most stable state where 5*s* and 5*p* electrons still untouched, acting to screen the energy levels of 4f electrons from the influence of external factors^11^. Glassy network is a good environment for impending such rare earth ions to enhance many optical, structure and mechanical advantages of the glasses where bonding connection’s becomes more ionic with adding rare-earth ions. In spite of the disadvantages of using such expensive rare earth oxides in preparing special types of glasses, this problem can be avoided by doping them in low concentrations and produce an extremely valuable economic product. For instance, the production of radiation shielding borosilicate glasses from useless waste material represents a newly interesting method to produce very economic and promising applicable material, particularly to achieve a dual target; getting rid of the wastes with their polluting hazards and production of an important useful product shelters radiation hazards.

The impact of ionizing gamma radiation on glass network commonly comprises the possible creating of defect color centers e.g., electron hole traps and/or non-bridging hole centers NBOH. Composition nature of the glass controls the rate of radiation effect on its structure for instance specific rigid and compacted glassy structures can avoid the passage of radiation photons revealing precious radiation shielding properties. Although many studies have studied the preparation of glasses from different wastes, it is a new topic to study the effect of irradiation on the optical features of such waste prepared glasses to magnify their applicable uses especially for radiation protection applications.

Herein the objective of the current work is the fabrication of new borosilicate glass systems from municipal solid waste (MSW) ashes doped with CeO_2_ and/or Gd_2_O_3_, and characterizing many of their structural, physical, optical, and shielding features regarding the impact of 100 kGy of gamma radiation in order to evaluate their practicable and promising use as radiation shielding protective materials.

## Experimental works

### Preparation

Glass specimens with the composition; (50 – x) MSW ash + 20 B_2_O_3_ + 20 SrO + 10 Na_2_O + X (wt%), where x = 0.7 CeO_2,_ 0.25 Gd_2_O_3_, or 0.5 CeO_2_ + 0.3 Gd_2_O_3,_ were prepared using the typical melting-quenching technique. MSW ash that used in making glasses was taken from landfill- waste region in the eastern Cairo. At the beginning, definite amounts of MSW ash were burned in an aching furnace (SNOL, 2/1100 LHM01 with fiber-insulated chambers, Russia.) at 750 °C for 3 h for completing volatility and breaking down of the residual organic materials. Using an automatic calibrated sensitive balance (four digits Kern analytical balance- Model ABJ 220-4M, Germany, accuracy ± 0.0001), and depending on XRF analysis of the burned MSW ashes listed in Table 1, accurate amounts of MSW ash were weighed and added to other weighed chemical materials consistent with glass compositions scheduled in Table 2. Chemicals added to the burned MSW ash were the purely chemical reagents from H_3_BO_3_, SrCO_3,_ Na_2_CO_3_, Gd_2_O_3_ and CeO_2_. The three batches were then mixed sensibly, grinded by a gate mortar and melted in platinum crucibles at 1450 °C in an electric muffle furnace for 2 h (Nabertherm muffle furnace, Capacity 8 L/17, Germany). The crucibles were revolved during melting process at diverse interval periods for homogeneity and removing air bubbles. After completing melting of glasses, the melts were molded on heated stainless steel molds (dimensions of 1 × 1 × 0.2 cm^3^). Formerly, the fabricated specimens were straightly conveyed to annealing furnace (Nabertherm, 15 L/12, Germany) for annealing samples at 550°C for 1 h, then the muffle was discontinued leaving the samples in, over the night till they reached room temperature by ± 25 °C/h cooling rate. Lastly, well-polished and finely crushed samples were prepared for measuring glass samples on various techniques.

**Table 1:** XRF analysis of the burned municipal waste ash (MSW).

Oxide	Na_2_O	MgO	Al_2_O_3_	SiO_2_	P_2_O_5_	SO_3_	CaO	TiO_2_	Cr_2_O_3_	MnO	Fe_2_O_3_	NiO	SrO	ZrO_2_	CdO	Cl
Concentration (wt%)	50.78	1.78	2.32	38.65	0.03	0.226	4.94	0.04	0.009	0.017	1.004	0.007	0.006	0.022	0.009	0.074

**Table 2:** Compositions of the prepared glasses (wt%).

Sample code	Waste	B_2_O_3_	SrO	Na_2_O	CeO_2_	Gd_2_O_3_	Density, g/cm^3^
S1 (RE1)	50-x	20	20	10	0.7	–	3.0087
S2 (RE2)	50-y	20	20	10	–	0.25	3.0078
S3 (RE3)	50-(x + y)	20	20	10	0.5	0.3	3.0100

### Measuring techniques

XRF Philips sequential (X-ray spectrometer-2400) was used to evaluate the chemical composition of the MSW ash. A batch of burned MSW ash was powdered finely and combined with H_3_BO_3_ as a binder for easier pressing before XRF measuring. Structures of samples were also designated via X-ray diffraction instrument (XRD) (Shimadzu, Japan with scanning rate; 8°/min and at 2ϴ range; 4°–90°). The optical properties were measured in a wavelength range 190–1000 nm using a double beam UV–Vis spectrophotometer, Unicam, England. Each glass sample was dignified three times to settle the accuracy of the absorption peaks. Photoluminescence measurements were carried out using a fluorescence spectrophotometer; xenon flash lamp JASCO, FP-6500, JAPAN, and the source of excitation light was utilized to measure photoluminescence at room temperature. FTIR technique was used to analyze the prepared glasses’ structures in a wavenumber range 400–3000 cm^−1^ by a FTIR spectrophotometer, type VERTEX 70, FT/IR-430. ESR was measured by X-band EMX spectrometer with a rectangular opening of the typical Bruker ER 4102-Germany. At lab-temperature, all ESR assessments were completed with a single scan (25 ± 2 °C).

Density (ρ) was calculated from the subsequent relation;

1 $$\rho = \left( {\frac{a}{a - b}} \right) \times 0.86\,\,\,\,{\text{g}}/{\text{cm}}^{{3}}$$

ρ: density of the glass sample, a and b: weights of glass samples in air and xylene, respectively, and 0.86: xylene density at 20 °C, densities was dignified three times to check measuring correctness by uncertainty values ± 0.002 g/cm^3^.

Microhardness of the prepared glasses was investigated by the Vickers hardness tester (Shimadzu HMV type, Japan) at specific applied loads of 100 g for 15 s to analyze microhardness. Microhardness examinations were measured three times to confirm the accuracy of measuring with an uncertainty value ± 0.46 kg/mm^−2^.

The Vickers hardness (HV) was calculated by the next equation:

2 $$HV= \frac{1.8544P}{{d}^{2}} (\text{kg} {\text{mm}}^{-2})$$

where (1.8544) is constant geometrical factor for diamond pyramid, (P) is an applied load in gram, and (d) is diagonal length of the indenter impression in mm.

Thermal expansion behavior of the synthesized glassy specimens was evaluated by a computerized dilatometer (Model NETZCH-Dil-402, Germany). Each sample was measured with the rate of 5 °C/min from RT up to the dilatometric softening temperature.

Irradiation process with a dose of 100 kGy, was taken place at 25 ± 5 °C, by the source of Co^60^ γ-cell-220 (made by the Indian Atomic Energy Authority) at National Center for Radiation Research and Technology (NCRRT), Egyptian Atomic Energy Authority (EAEA).

Using a user-friendly online Phy-X/PSD tool, the produced glasses' radiation shielding capabilities are evaluated to calculate many important shielding parameters such as^12^, the major radiation shielding parameter, mass attenuation coefficient (MAC = µ/ρ), that can be obtained by means of chemical compositions and densities of the glass samples shown in Table 2 as^12–17^:

3 $$\frac{\mu }{\rho } = \mu_{m} = \sum\limits_{i} {W_{i} \left( {\frac{\mu }{\rho }} \right)_{i} }$$

µ: linear attenuation coefficient (LAC = µ = MAC* ρ) for the glass specimen, formerly numerous radiation protection factors were assessed such as^12–18^:

(i) The half value layer (HVL, T_1/2_):

4 $$HVL = T_{1/2} = \frac{0.693}{\mu }$$

(ii) Mean free path (MFP, λ):

5 $$MFP = \lambda = \mu^{ - 1}$$

(iii) Effective atomic number (Z_eff_):

6 $$Z_{eff} = \frac{{\sum\nolimits_{i} {f_{i} A_{i} \left( {\frac{\mu }{\rho }} \right)_{i} } }}{{\sum\nolimits_{j} {\frac{{A_{j} }}{{Z_{j} }}\left( {\frac{\mu }{\rho }} \right)_{j} } }}$$

where A_i_ and f_i_ are atomic mass and molar fraction of pure elements for each glass. 

### Ethical approval

The study included neither human or animal experiments, and no ethics permission was needed.

## Results and discussion

Table 1 revealed XRF analysis of the burned MSW ash that was used in preparing glasses where it was the source of SiO_2_ in such borosilicate glass systems because of containing about 38.65% of silica content. Table 2 revealed the whole chemical compositions of the prepared glass systems in wt%.

**Figure 1:**
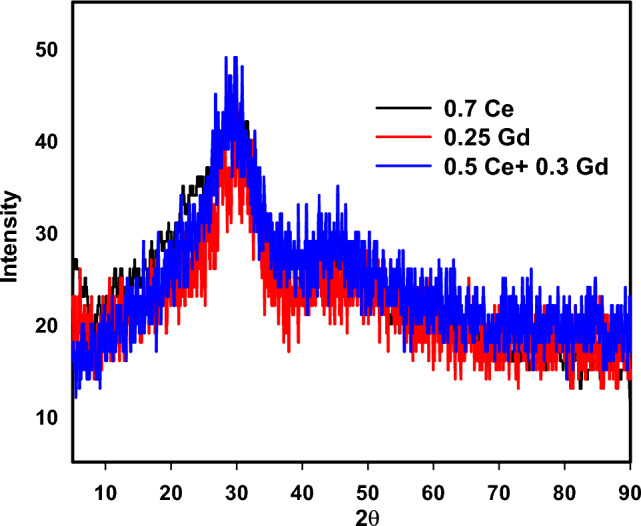
XRD pattern of the prepared the three RE borosilicate glasses.

Figure 1 pattern revealed the XRD spectra of the three fabricated RE-borosilicate glasses. As noticeable, broad hump peaks and the whole lack of crystalline peaks, indicate the amorphous and disordered structures of the prepared samples and then their glassy natures.

### Optical UV–visible measurements

The glass structure is highly favored as a host medium for rare earth ions because of their heterogeneously widened line widths of optical conversions and the simple compositional change. Lanthanides or RE ions are characterized by their electronic configuration [Xe] 4*f*^12^6*s*^2^; they have the outer-shell configuration of 4f^n^5s^2^5p^6^6s^2^, with partially filled 4f shell^11^. So their optical transitions in glasses are resemble predominantly to intra f^N^ transitions of the primarily electric dipole oddity and their noticeable absorption peaks are detected from infrared to ultraviolet regions. While visible and near IR absorption fit to the transitions between f–f energy levels however, UV absorption is produced by the movement from the low energy level 4f to the high energy level 5d^19^.

**Figure 2:**
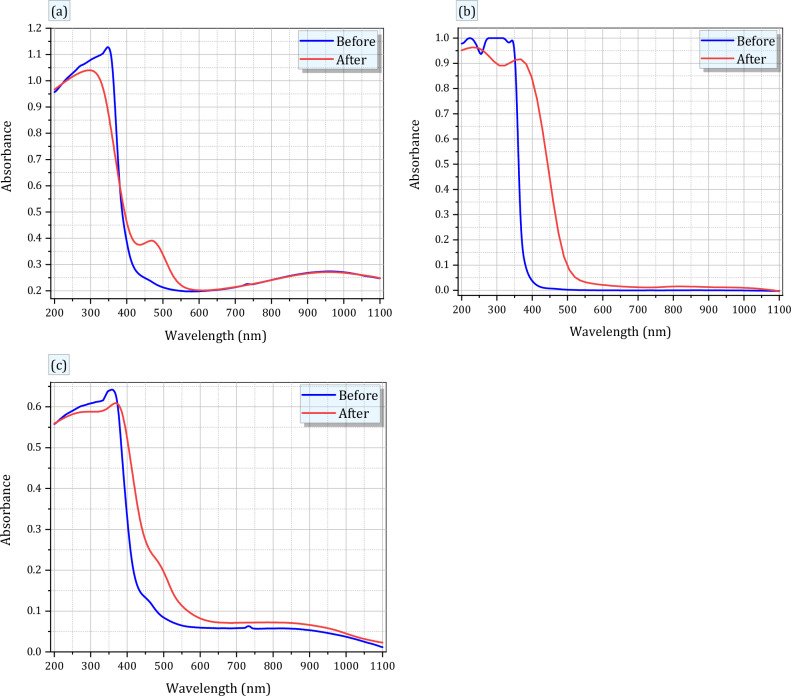
Optical absorbance of RE-glasses before and after 100 kGy of gamma irradiation for (**a**) RE1(Ce) (**b**) RE2 (Gd) and (**c**) RE3(Ce + Gd).

Figure 2a–c presents UV-near infrared absorption spectra of the fabricated RE (Ce, Gd and Ce + Gd) doped borosilicate glasses before and after gamma irradiation. As offered in Fig. 2a, the absorption spectrum of Ce doped glass mainly contains absorption bands as follows; a main peak at 351 nm (UV-regions), cutoff peak at 430 nm (Vis-regions) and wide broad band at 950 nm (NIR-regions). The main UV peak observed at 351 nm originates from the absorption of Ce^3+^ with 4f → 5*d* transitions^20^, where, the absorption of Ce^3+^ occurs in the higher energy region, while the charges transfer absorption of Ce^4+^ occurs in the lower energy region.

Also, from Fig. 2b, the absorption spectrum of Gd doped glass revealed obvious peaks at about 230, 270 and 348 nm allocated to the transition of 4f → 5d in Gd^3+^ ion^20^. As known there are two steady oxidation statuses for gadolinium; Gd^3+^ and Gd^4+^, having f1 and f^0^ electronic configurations, respectively^20,21^. The absorption of Gd^3+^ ions has the allowed transition (f^1^ → d^1^) that performs as broad and exhaustive absorption bands in several solid environments and depends intensely on the host composition and appears usually in UV region in most oxides. While, absorption bands correlated to Gd^4+^, are caused by the charge transfer nature O_2_ → Gd^4+^, that typically arises at the longer wavelength comparing to that of Gd^3+^^21,22^.

Moreover, Fig. 2c shows also the absorption of a mixed RE Ce and Gd doped borosilicate glass, where the absorption spectrum contains weaker absorption bands than the glass doped only Ce or Gd ions, separately. Additionally, the detected absorption peaks for Ce + Gd doped glass are at ~ 370 nm in UV-region can be ascribed to the presence of Ce within the glass composition rather than Gd. The small red shift of UV peaks detected at 351 and 348 nm in Ce and Gd doped glasses, respectively, is directly correlated to the small difference in the concentrations of both Ce and Gd ions and the expected electric dipole allowed 4f → 5d transitions for Ce and Gd ions^19^.

Figure 2 depicts also the UV–VIS-NIR absorption spectra of the same specimens after being exposed to 100 kGy of gamma irradiation. From Fig. 2a–c, all the glass samples displayed a minor reduction in their UV absorption bands. This reduction in optical absorbance was accompanied with a red shift in the fundamental absorption edges. The impact of ionizing radiation such as gamma rays on materials might occur some photochemical reactions e.g., redox reactions accompanying with variations in oxidation states of doped ions^23,24^.The obvious decrease in absorbance intensity indicates the optical stability of the glasses towards irradiation, while the clearness of secondly peaks at 480, 430 and 500 nm for Ce, Gd and Ce + Gd doped glasses, respectively can be attributable to a possible crystal field splitting of 5d (2p) state in Ce and Gd ions^25^. Some authors^19,26^ stated that the coexistence of several peaks in Ce doped glass is recognized to the non-equivalence of Ce ions, such as the change in coordination number with the adjacent ligands from one situate to another leading to an inhomogeneous broadening of optical peaks.

To quantify the impact of γ-radiation on the optical features of the investigated glasses, the optical gap (${E}_{g}$) can be considered from Mot and Davis model^27^ according to the following relation;

7 $$\alpha h\nu = B \left( {h\nu - E_{g} } \right)^{{\text{m}}}$$

**Figure 3:**
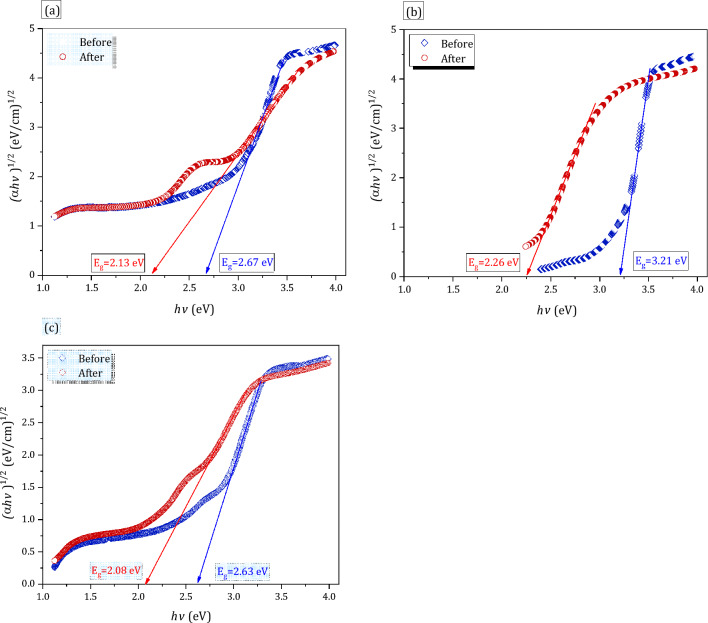
Plot of (αhν)^1/2^ against hν (Tauc’s plot) for RE-glasses before and after 100 kGy of gamma irradiation for (**a**) RE1(Ce) (**b**) RE2 (Gd) and (**c**) RE3(Ce + Gd) doped glasses.

**Figure 4:**
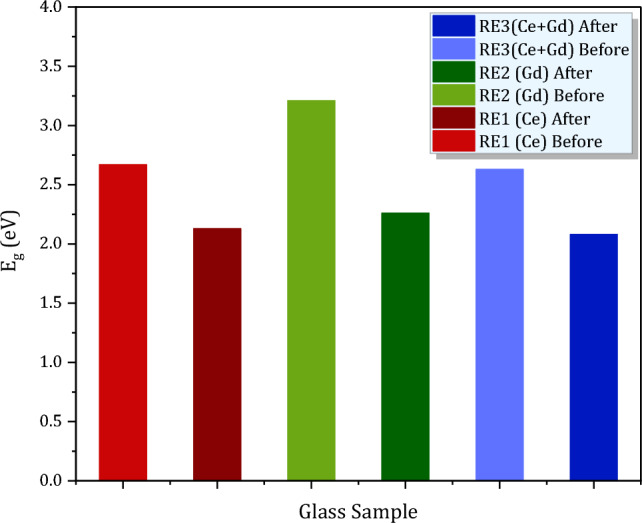
The optical band gap before and after 100 kGy of gamma irradiation for RE1(Ce), RE2 (Gd) and RE3(Ce + Gd).

**Figure 5:**
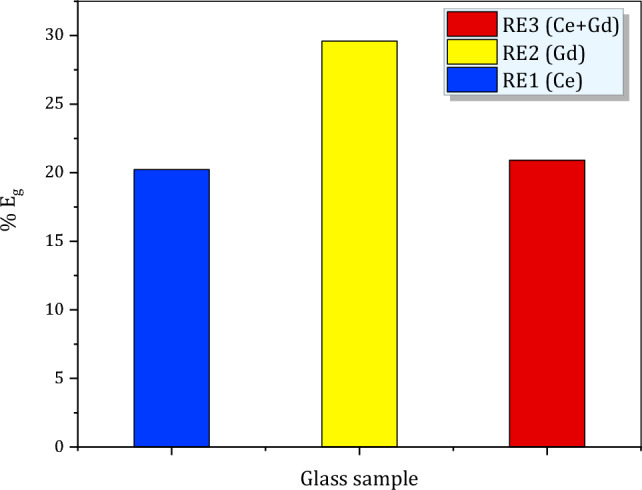
Percent of change in the optical band gap (% *E*_*g*_) for RE-glasses after 100 kG of gamma irradiations.

Herein, $h\nu$ is photon energy and Fig. 3a–c shows Tauc’s plots (at which we draw (*αhν*)^1/2^ versus *hν*)^27^) for (Ce, Gd and Ce + Gd) doped glasses before and after 100 kGy of gamma irradiation and obtained values of *E*_g_ are displayed in Fig. 4. From Fig. 4 we can easily observe the slight reduction of *E*_g_ values for the three glass samples after being irradiated with 100 kGy of gamma irradiation. Furthermore, the percent of *E*_g_ changes ($\text{\% }{E}_{g}$) was employed here to assess the RE-glass stabilities under gamma radiations according to the relations;

8 $$\text{\% }{E}_{g}=\frac{{E}_{g0}-{{E}_{g}}_{100}}{{E}_{g0}}\times 100$$

where ${E}_{g0}$ and ${{E}_{g}}_{100}$ represent the band gap for the un irradiated glass samples and 100 kGy irradiated glass samples. The $\text{\% }{E}_{g}$ values for all glasses are exhibited in Fig. 5. From the figure it is noticeable that $\text{\% }{E}_{g}$ is the smallest for Ce doped glass sample (20.2%) and the largest for the Gd glass sample (29.6) and in between for Ce + Gd doped glass sample (21%). From these values we can deduce that, the Ce-doped glass sample has the highest optical stability against gamma irradiation.

### Photoluminescence measurements (PL)

**Figure 6:**
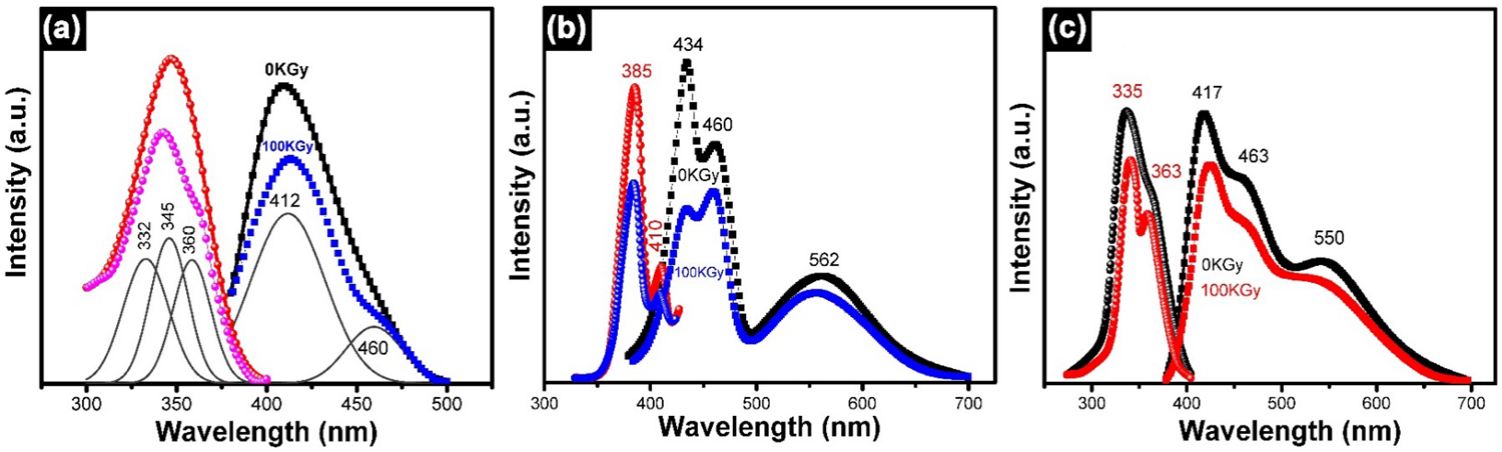
The photoluminescence spectra of RE doped-glasses before and after 100 kGy of gamma irradiation where (**a**) 0.7% CeO_2_ (**b**) 0.25 Gd_2_O_3_ and (**c**) 0.5%CeO_2_ + 0.3Gd_2_O_3_.

The results of photoluminescence measurements on CeO_2_ and/or Gd_2_O_3_ doped glasses are shown in Fig. 6. As illustrated in Fig. 6, the excitation spectra of all samples are located in the UVA spectral range, approximately at about 332–385 nm. An observable small sharp excitation peak at about 410 was characteristic of Gd_2_O_3_-doped glassy sample. The fluctuation in excitation energy is caused by the nature and concentration of the co-doped rare earth ions. Glasses can contain cerium in the polyvalent states of Ce^3+^ and Ce^4+^, whose balance or proportion varies depending on the host glass's type and composition, temperature of melting and atmosphere^28^. For the first sample shown in Fig. 6a, the excitation spectrum may be due to the occurrence of mutually two cerium valences within the investigated glasses, and an asymmetric broad excitation at about 332–360 nm identifies the trivalent transition (Ce^3+^ ion) 4f → 5d of Ce^3+^ ion agreed with the optical spectra shown in Fig. 2a for Ce doped glass.

Cerium is a fascinating element that often exhibits remarkable luminescent properties when incorporated into host materials. The emission spectra of cerium-doped materials can provide valuable insights into its electronic structure and the intricate interactions it has within the host lattice. Cerium can exist in different oxidation states, and the electronic configuration of Ce^4+^ is particularly interesting. Unlike Ce^3+^, which has a 4f^1^ configuration and displays strong 5d-4f transitions, Ce^4+^ has a 4f^0^ electronic configuration, meaning it lacks any 4f electrons. This absence of 4f electrons significantly influences the luminescence properties of Ce^4+^. In this case, the luminescence observed in Ce^4+^doped materials arises from charge transfer transitions, typically from the valence band or ligand orbitals to the empty 4f orbital of Ce^4+^. These transitions are often referred to as ligand-to-metal charge transfer (LMCT) transitions. The study of these LMCT transitions in Ce^4+^doped materials can provide valuable insights into the electronic structure and interactions within the host lattice, offering a deeper understanding of the fundamental properties of this fascinating element.

According to Fig. 6b, the excitation spectra of Gd^3+^ doped glass reveal two characteristic excitation bands that can be allied to transitions from the ground state of Gd^3+^ ion to the higher ^8^S_7/2_–^6^P_7/2_, ^6^I_J_, and ^6^D_J_ excitation states^29^. For the mixed rare-earth doped glass, the spectral excitation bands are located sharply at 335 nm with an extended band at 363 nm as shown in Fig. 6c. The emission spectra of the three samples depicted an intense bands centered at 412, 434, and 417 nm with a fixed extended emission band centered at about 360 nm for CeO_2_, Gd_2_O_3_, and (CeO_2_ + Gd_2_O_3_) doped glasses, respectively. The emission of pure Gd_2_O_3_- doped sample shows a characteristic emission band centered at about 562 nm and when mixed with CeO_2_ the emission band shows a minor shifting to smaller wavelength at 550 nm. Consequently electronic transitions from the excited (5d) to (4f) ground statuses, trivalent cerium ions produce an emission spectrum that is split into two energy sublevels such as ^2^F_7/2_ and ^2^F_5/2_, and to identify the sub-peaks originating from 5d → ^2^F_7/2_ and ^2^F_5/2_ transitions. It is determined that the amount of Ce^3+^ ions present affects the relative emission intensity of the glass^30,31^. Kumar et al.^32^ proposed that, when gadolinium is utilized as a dopant, the energetic levels of Gd^3+^ ion contain several transitions detected as a simultaneous fluorescence analogous to transitions ^4^F_(4)J_ → ^6^G_J_, ^6^G_J_ → ^6^P_J,_ and ^6^P_J_ → ^8^S_7/2_ centered nearby 826, 601 and 313 nm, respectively^32^. The proposed hypothesis by Mal’chukova et al.^29^ assumed that the matrix's energy may migrate to the Gd^3+^ ion's ^6^D_J_ levels and then non-radiatively relax to the ^6^P_7/2_ level. With a subsequent emission, the energy of the Gd^3+^ ion may likewise shift from the ^6^D_J_ (or ^6^P_J_) levels to the levels of defects^29^. The photoluminescence spectra appearing in Fig. 6c are strong indication that efficient energy transfer from Gd^3+^ to Ce^3+^ should occur. Depending on the emission spectrum of the third rare earth mixed glassy sample that can be attributable to charge transfer bands, ^8^S_7/2_ → ^6^I transitions of Gd^3+^ ions, and 4f → 5d transitions of Ce^3+^ ions. The Gd^3+^ ions can be excited to ^6^I, and then quickly return to ^6^P_7/2_ which can transported to the 5d levels of Ce^3+^ by resonant energy transfer, improving the luminescence of Ce^3+^^33^. The energy transfer process in the glass co-doped Gd^3+^ and Ce^3+^ ions, is illustrated by additional important energy levels of Gd^3+^ and Ce^3+^ ions that Wang et al.^33^ detected. According to this diagram of energy levels, energy can be transferred from the ^6^P, ^6^I, and ^6^D states of Gd^3+^ to the 5d state of Ce^3+^^31,33^.

Upon gamma irradiation, the intensity of the photoluminescence spectra is slightly reduced. The effect of gamma irradiation can be initiated by a number of different processes or mechanisms, including charge trapping, radiolysis, photochemical reaction, rupture, atomic displacement, or reformation of some connecting bonds^28,30^. The effects of one or more of the previously mentioned processes may be related to the alterations created by gamma radiation, but only to a limited extent due to specific shielding behavior resulting from the glass composition^28,30^. Although, the lower intensity of the PL spectra may be correlated to coloration or formation of induced defects that occur in the glassy shape that may lead to variations in the bond angles and/or lengths of specific structural groups and affect both intensities and positions of the emission peak, the reduction in absorbance intensity shown before in Fig. 2 agreed with PL spectra of the glasses indicating the possible relaxation in the glasses’ optical properties after irradiation.

**Figure 7:**
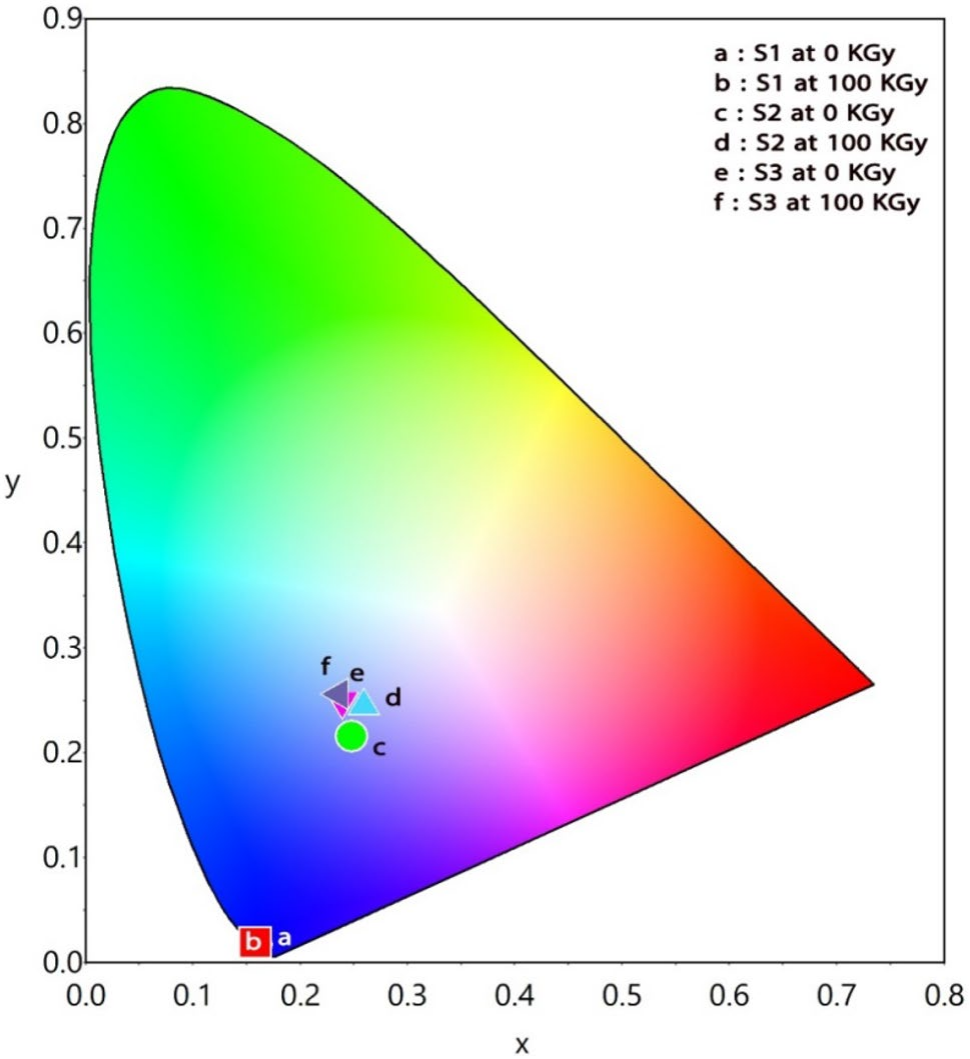
CIE -chromaticity coordinates of the prepared glassy samples.

Figure 7 displays the CIE chromaticity diagram of the prepared CeO_2_ and/or Gd_2_O_3_ doped glasses denoted by S1, S2, and S3. A complete set of CIE color coordinate values can be found in Table 3. The color coordinates are equally distributed and centered in the blue region. The interpretation of the observed colors is based on the presence of rare-earth dopants that can emit blue hues, and the protracted appearance of various blue degrees is related to the type of dopant in the host glass matrix as a whole. For CeO_2_–doped glass, the deep blue coloration shared the areas (a) and (b) of Fig. 7. The rest of the areas (c,d,e, and f) are located in the brightest blue area with the addition of Gd^3+^ ions. This suggests that Gd^3+^ ions increase Ce^3+^ ions luminescences in certain materials and more effectively transmission energy to Ce^3+^^33^.The optical absorption spectra with other doped nano-CeO_2_ doped silicate glasses revealed a characteristic peak of Ce^3+^ ions that generates a yellowish appearance^34^, while a mix of blue, yellow, and red bright yellow emissions were produced for Gd/Dy (at 576 nm) in Gd_2_O_3_-B_2_O_3_ glasses^35^.

**Table 3:** CIE chromaticity coordinates of S1, S2, and S3 glasses before and after 100 kGy of gamma irradiation.

Irradiation dose	Sample code					
	S1(CeO_2_)		S2(Gd_2_O_3_)		S3(CeO_2_ + Gd_2_O_3_)	
	x	y	x	y	x	y
0 kGy	0.159	0.017	0.248	0.216	0.239	0.252
100 kGy	0.158	0.019	0.259	0.246	0.236	0.256

### FTIR absorption spectra

**Figure 8:**
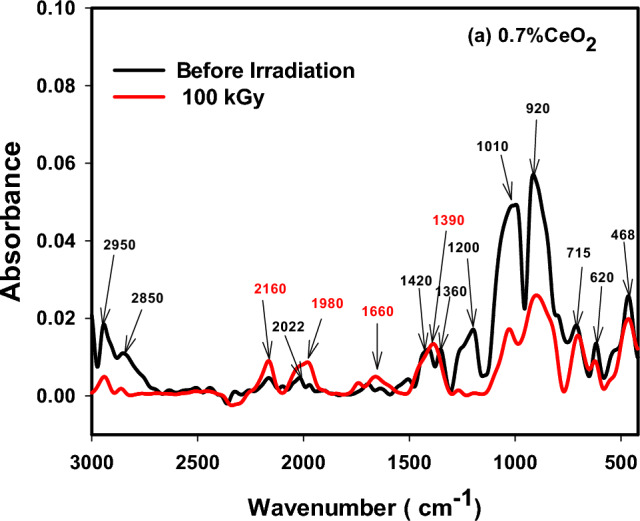
FTIR absorption spectra of CeO_2_ doped borosilicate glass before and after 100 kGy of gamma radiation.

**Figure 9:**
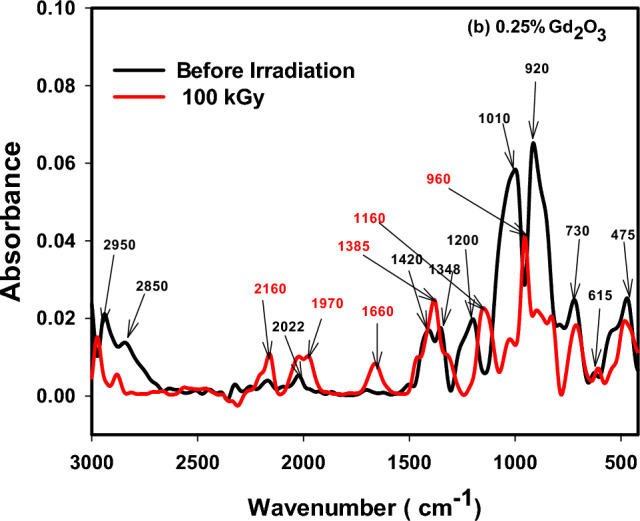
FTIR absorption spectra of Gd_2_O_3_ doped borosilicate glass before and after 100 kGy of gamma radiation.

**Figure 10:**
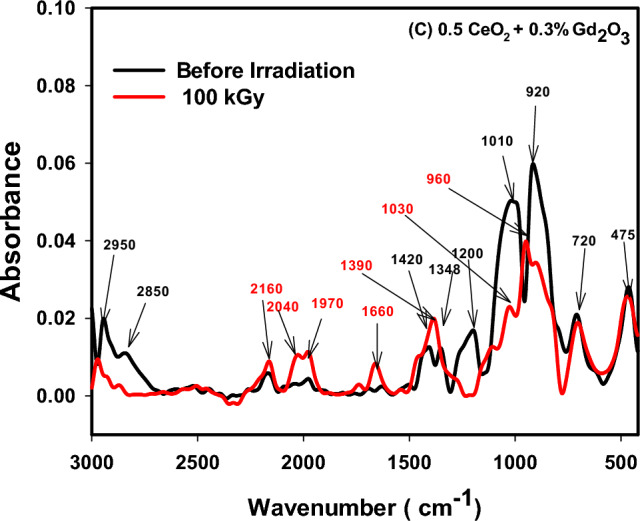
FTIR absorption spectra of CeO_2_ + Gd_2_O_3_ doped borosilicate glass before and after 100 kGy of gamma radiation.

The various building units of the present borosilicate structure was identified by FTIR spectroscopy that involves predominantly the trigonal building units of borate BO_3_, and tetrahedral building groups of silicate, SiO_4_ and borate BO_4_. Figures 8, 9 and 10 displayed FTIR absorption spectra of the three deliberated borosilicate glass systems before and after 100 kGy of gamma irradiation. The three figures revealed similar spectra before irradiation showing an intersecting between silicate and borate vibrational bands. The spectral features and their assignments in the three spectra before and after irradiation can be discussed as follows;

(a) Before irradiation.

1. The small bands appeared at 468 or 475 cm^−1^ can be ascribed to the vibrational signals of the present modifier cations into residing locations shown in Tables 1 and 2 such as the transition metal (TM) ions e.g., Cr^3+^, Ti^4+^, Fe^3+^, Cr^3+^ and Mn^2+^ as well as the vibration of alkali ions such as Na^+^, Mg^2+^, Ca^2+^ and Sr^2+^ions.

2. The band appeared at 620 or 615 cm^−1^ interrelated to the silicate and borate bending mode vibrations, in addition the vibration of compacted units of aluminum ions as AlO_4_ or AlO_6_^6,36^ as well as a possible combined vibrations of the tetrahedral units; AlO_4_, SiO_4_ and BO_4_ units^5,6^.

3. The band appeared at 715, 720 or 730 cm^−1^ recognized to silicate bending mode vibration (Si–O–Si or O–Si–O), or bridging oxygens (BO) bending vibrations of borate amid two trigonal boron atoms (B–O–B linkages) and a possible Si–O–B link bending vibrations^5,6,37^.

4. A main band appeared at 920 cm^−1^ correlated either to Si–O–NBO stretching vibration, or vibrations of numerous borate groups (tri, tetra or penta borate) and B–O–M linkages’ stretching vibrations (M signifies metal ions or diborate group)^38^.

5. A main larger band appeared at 1010 cm^−1^ assigned to mutual Si–O–Si and B–O–B stretching vibrations in tetrahedral BO_4_ building units.

6. A small band appeared at 1200 attributed to tri, penta, or tetraborate boroxal groups’ stretching vibrations^39^.

7. Moderate bands appeared at 1348 or 1360 and 1420 cm^−1^ can be attributed to the asymmetric stretching vibrations of B–O bonds in the trigonal borate groups [BO_3_], [BO_2_O]^−^, BO_3_ with non-bridging oxygen (NBO)^36,38^.

8. Bands appeared from 1600 cm^−1^ up to the spectrum end at 3000 cm^−1^ recognized to H-bonding, molecular water, OH^−^, Si–OH and B–OH stretching vibrations that are not dominant glass forming units^5,6,40^.

As obviously shown the three spectra are very similar before irradiation because of the similar composition and the small doping contents of the added rare earth ions 0.7 Ce, 0.25 Gd or 0.5 Ce + 0.3 Gd ions.

(b) After 100 kGy of gamma radiation.

Figures 8, 9 and 10 revealed two core observations after samples’ irradiation with 100 kGy of γ-rays. The first is the obvious decrease in absorption intensity of the spectra after irradiation specially the two main bands that appeared before irradiation at 920 and 1010 cm^−1^, and the second is shifting the band at 920 cm^−1^ to high wavenumber at 960 cm^−1^. The two observations are directly interrelated to more stable structures where the lower absorption intensity refers to the release of extra energy and possible formation of relaxed structures. However, the shift of the band from 920 to 960 cm^−1^ and the slight increase in absorption intensity of the non-essential groups ranged from 1600 to 3000 cm^−1^ can be assigned to a quite adjustment in the polymerization degree^37,41^. Mostly, the three spectra revealed the same effect towards irradiation as they give approximately more relaxed structures, indicating the negligible irradiation influence on their rigid structures. This manner is governed by the valuable compositions of the fabricated glasses, as shown in Tables 1 and 2, where the elemental rich composition of the MSW ash that utilized chiefly in glasses’ preparation—as listed in Table 1—is the main reason for their stability against the influence of ionizing radiation. The following arguments elucidate in detail the helpful effect of glass composition on its structural stability against irradiation;

(c) The dual behavior of the high compacted silicate tetrahedral (SiO_4_), trigonal (BO_3_) and tetrahedral borate (BO_4_) building units, strengthens the glassy network to resist the consequence of radiation photons.

(d) Presence of different transition metal ions in the used MSW ash as listed in Table 1 (e.g., Mn^2+^, Cr^3+^, Ti^4+^, and Fe^3+^) plays an important role in absorbing possible defects that may be caused by irradiation, due to their ability to change their outermost configurations and coexistence in different oxidation states.

(e) The effect of the doping rare earth ions such as CeO_2_ and Gd_2_O_3_ support borosilicate glass resistance to irradiation ascribed to their remarkable mass attenuation owing to their large atomic number, especially cerium ions that participate in the glassy system in both valence states of ;Ce^4+^ and Ce^3+^^42,43^.

### Electron spin resonance ESR

**Figure 11:**
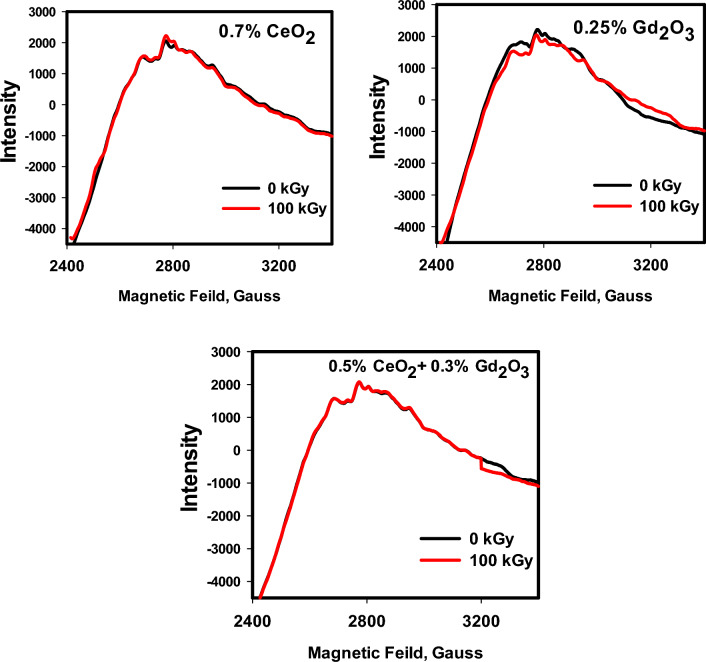
ESR spectra of the three prepared glasses Ce (RE1), Gd (RE2) and Ce + Gd (RE3), before and after 100 kGy of gamma radiation.

ESR technique was used to recognize chemical species that contain one or more free electrons and identify changes in paramagnetic centers formed because of irradiation processes. In the amorphous glassy network, these defect centers may be positive holes and/or electron defect centers^2,6^. Figure 11 reveals ESR spectra of three prepared RE borosilicate glasses before and after 100 kGy of γ-radiation. Figure 11 shows the high similarity between the three studied glasses either in positions or intensity. The three ESR spectra revealed the same spectra before and after irradiation with 100 kGy besides the absence of real sharp peaks, signifying the trivial effect of irradiation on the glass structures and no formation of free radicals^2^. Consequently, the high bonding connectivity of the glasses because no breaking in bonds was taken place and thus no free radical was formed under the effect of irradiation. The stable ESR spectra can be correlated also to stability in optical spectra and the relative stability in FTIR spectra after irradiation. This large stability in glasses properties are owing to the rigid structure of borosilicate network that consists of the tetrahedral SiO_4_, trigonal BO_3_ and the tetrahedral BO_4_ building units, so that they work together to block the path of radiation photons^44^. Subsequently, the high resistance of the considered glasses to ionizing gamma radiation and formerly their potential use for radiation protection applications.

### Physical and mechanical measurements

Table 4 displays the microhardness values for 0.7 CeO_2_, 0.25 Gd_2_O_3_, and (0.5 CeO_2_ + 0.3Gd_2_O_3_) doped borosilicate glasses, respectively, having values of 473, 492, and 510 kg/mm^2^. The observed progressive increment in the hardness values are interrelated to the change in the main glassy structure as a result of rare-earth addition. It can be associated to the changes in lengths or strengths of bonds of the glassy network due to the additional effects of CeO_2_ and Gd_2_O_3_. These data demonstrate that in the current glasses, CeO_2_ and/or Gd_2_O_3_ act as a glass modifier allowing the excess NBO formation that primes an increase in the glassy network firmness giving better and resilient bonding, so the glass exhibits higher hardness^45^.

**Table 4:** Hardness (H_V_), transformation temperature (T_g_), dilatometric softening temperature (T_d_), and thermal expansion coefficient (CTE) before and after 100 kGy of gamma irradiation.

Measurement	S1 (CeO_2_)		S2(Gd_2_O_3_)		S3 (CeO_2_ + Gd_2_O_3_)	
	0 kGy	100 kGy	0 kGy	100 kGy	0 kGy	100 kGy
H_v_ (kg mm^−2^) ± 0.46	473	478	492	501	510	518
T_g_ (°C) ± 3.6	493	503	468	485	493	520
T_d_ (°C) ± 2.5	535	541	505	518	536	552
CTE (*10^–6^/°C) ± 0.12	11.55	11.73	17.80	18.54	9.36	10.20

**Figure 12:**
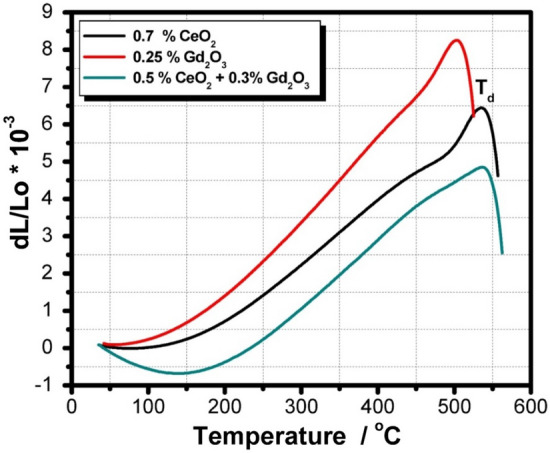
Thermal expansion curve of the prepared CeO_2_ and/or Gd_2_O_3_-doped glasses before gamma irradiation.

Figure 12 depicts the thermal expansion curve of the CeO_2_ and/or Gd_2_O_3_-doped glasses before irradiation. Glass transformation temperature (T_g_), dilatometric softening temperature (T_d_), and thermal expansion coefficient (CTE) data are also itemized in Table 4. It is perceived that CeO_2_-doped glass reveal higher dilatometric softening temperature and thermal expansion coefficient compared with the Gd_2_O_3_- doped glass sample. A few of the factors influencing CTE, T_g_, and T_d_ of glasses such as the type of chemicals constituting the glass, bond strength, and the field strength of the cations^46,47^. According to the structures of the current glasses, adding CeO_2_ causes a drop in the thermal expansion coefficients while a rise in the glass transformation temperature (T_g_), and dilatometric softening temperature (T_d_). These occurrences are thought to be related to the creation of more NBO, which is what causes cerium ions to disrupt the network structures of glasses and reduce their connection^48,49^.

**Figure 13:**
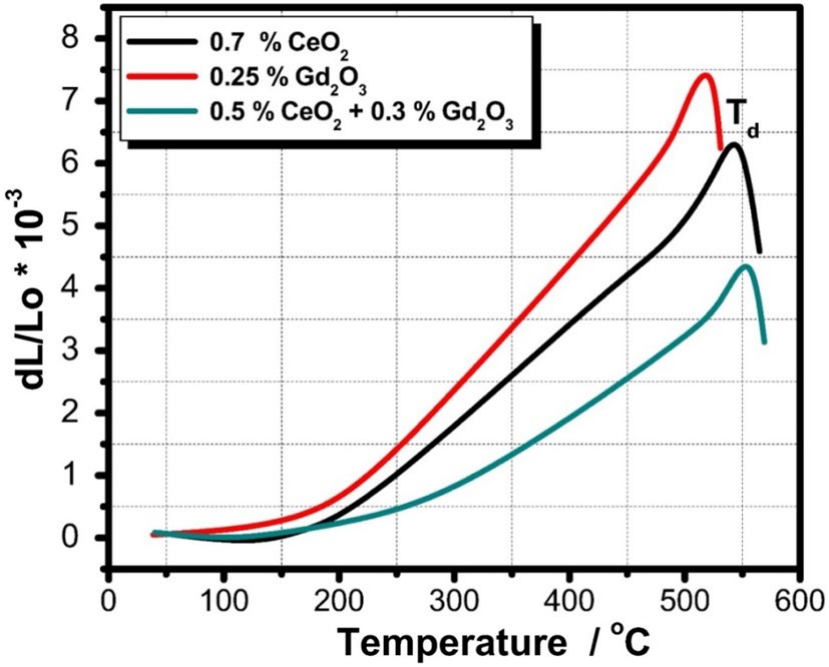
Thermal expansion curve of the prepared CeO_2_ and/or Gd_2_O_3_-doped glasses after 100 kGy of gamma irradiation.

It is generally accepted by many previous studies^49–51^ that the ionic radius and cation field strength of rare-earth ions are often the two factors that have the most influence on the action of rare-earth oxides in produced glass. Due to the strong field and excellent coordination of Ce^4+^ ions, CeO_2_ plays an important role in the mechanical and thermal behavior of the glasses^49–51^. On the other side Fig. 13 reveals the thermal expansion curve of CeO_2_ and/or Gd_2_O_3_-doped glasses after 100 kGy of gamma irradiation, revealing a large thermal stability of the glasses towards ionizing radiation.

### Radiation shielding characteristics

The three RE borosilicate glasses' potential for ionizing radiation protection has been reached by examination and regulation of the mass (MAC = µ/ρ), and linear (LAC = µ) attenuation coefficients. MAC and LAC mostly influenced by the properties of materials (in this case, the glass compositions) and the incident photon energy (E), rather than materials’ thickness. Photoelectric effect (PE), Compton Scattering (CS), and Pair Production (PP) processes are three examples of photon interaction mechanisms that are taken into account while calculating the MAC physically^52–55^.

**Figure 14:**
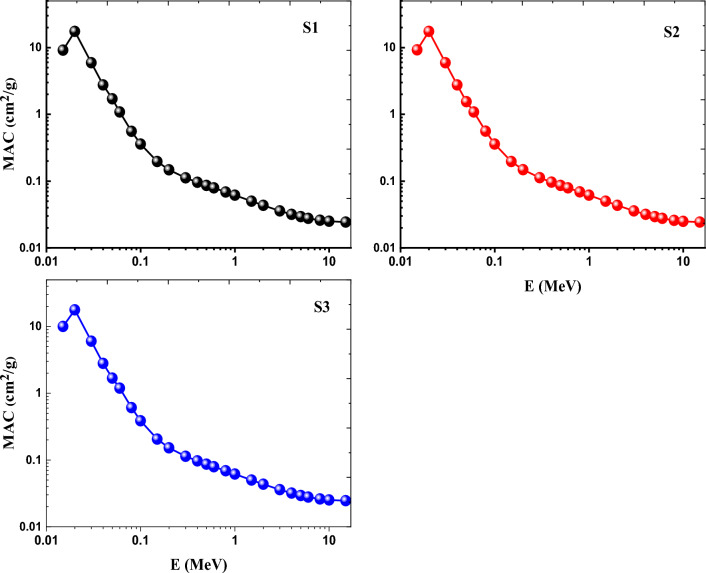
Variation of MAC as a function of photon energy of Ce (S1), Gd (S2) and Ce + Gd (S3) doped borosilicate glasses.

Using the user-friendly online Phy-X/PSD tool, the MAC of the currently constructed glasses was assessed as a function of photon energy (E) in the range of 0.015 to 15 MeV^12^. The weight fraction of the elemental composition and the density of glasses were used in these computations. Figure 14 depicts the variance in MAC values for the inspected glasses as a function of photon energy (E) in MeV. Figure 14 illustrates how the inclusion of CeO_2_, Gd_2_O_3_, or combined CeO_2_ and Gd_2_O_3_ has a direct helpful impact on the MAC values. For all photon energies, it was found that the S3 sample doped with CeO_2_ and Gd_2_O_3_ had the highest MAC values compared to S1 sample doped with CeO_2_ alone and the S2 sample doped with Gd_2_O_3_. The highest MAC values for the S1, S2, and S3 samples were 9.239, 9.166, and 10.016 cm^2^/g at 0.015 MeV respectively. The lowest MAC values for S1, S2, and S3 were, respectively, 0.024 cm^2^/g, 0.024 cm^2^/g and 0.025 cm^2^/g at 15 MeV. The behavior of the MAC typically follows the hierarchy (S3)_MAC_ > (S1)_MAC_ > (S2)_MAC_. The following are some explanations for the MAC of all glasses trend: The photoelectric effect (PE) cross section varies directly with Z^4^ E^−3^ (Z is the atomic number of the absorbing material) at the lowest photon energy (E), as shown by the MAC's trend. The Compton Scattering (CS) cross section varies straightly with (Z/A) and oppositely with E in the intermediate zone of the photon energy. The cross section of pair production (PP) interaction varies with Z^2^ in the highest photon energy zone. In conclusion, the greater (Z) and denser the absorber, the higher the MACs have values. As a result, S2 glass with the lowest density (3.0078 g/cm^3^) and S3 glass with the highest density (3.0100 g/cm^3^) had the lowest and the highest MAC values, respectively.

**Figure 15:**
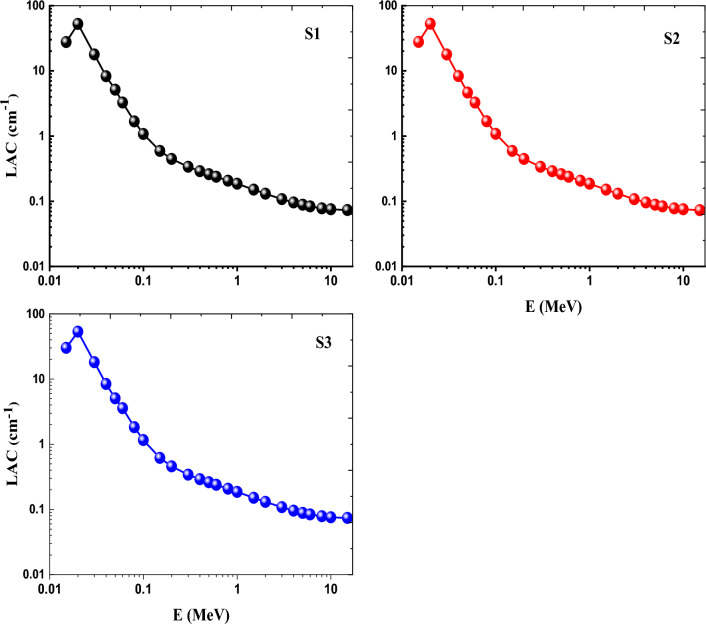
Variation of LAC as a function of photon energy of Ce (S1), Gd (S2) and Ce + Gd (S3) doped borosilicate glasses.

The fluctuation of the created glasses' linear attenuation coefficient (LAC) in cm^−1^ as a function of photon energy (E) is displayed in Fig. 15. The curves in Fig. 15 show that the LAC for all glasses follows a similar pattern, with S2 glass having the lowest values and S3 glass having the maximum values. Consequently, the LAC inclination moves in the following order: (S3)_LAC_ > (S1)_LAC_ > (S2)_LAC_.

**Figure 16:**
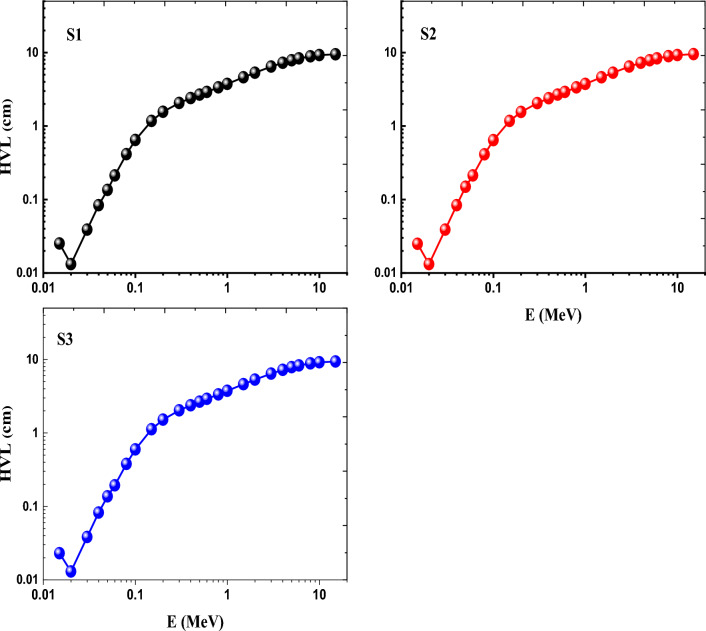
Variation of HVL as a function of photon energy of Ce (S1), Gd (S2) and Ce + Gd (S3) doped borosilicate glasses.

**Figure 17:**
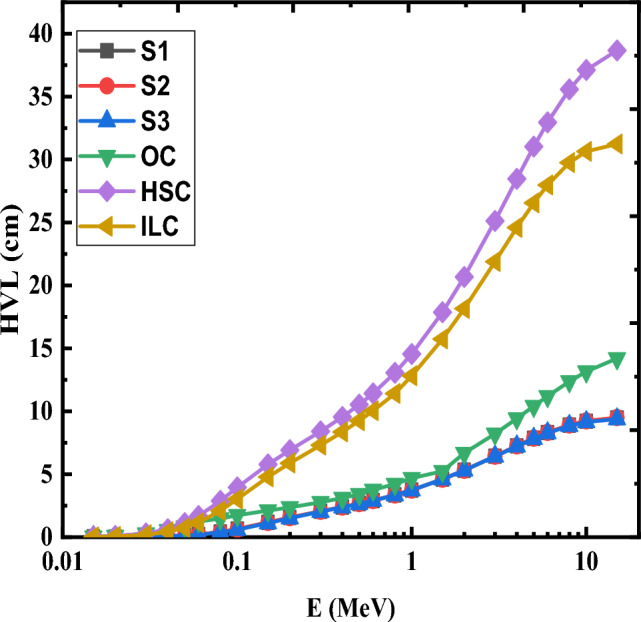
HVL of the prepared glasses compared with some commercial concrete.

In relation to the half-value layer (HVL), Fig. 16 shows how the HVL changes in cm with photon energy (E) of the produced glasses. The variance of HVL is minimal, and their values incline to be near together, in accordance with the dominance of (PE) cross sections in the low photon energy area. Attributable to the prevalence of both (CS) and (PP) interactions processes, the HVL raised and the values varied more between samples in the middle photon energy zone. While S3 glass, which has a high density of 3.0100 g/cm^3^, performed the deepest HVL values and changed from 0.023 cm at 0.015 MeV to 9.384 cm at 15 MeV, S2 glass, which has a low density of 3.0078 g/cm^3^, performed the highest HVL values changes from 0.025 cm at 0.015 MeV to 9.478 cm at 15 MeV. As a result, the HVL of the produced glasses behaves differently from the MAC and LAC. Consequently, (HVL)_S2_ > (HVL)_S1_ > (HVL)_S3_. The HVL results show that among all the examined glasses, S3 glass provides the best radiation protection. Figure 17 compares three samples of marketable concrete materials (Ordinary concrete (OC), Hematite-Serpentine concrete (HSC), and ILmenite-Limonite concrete (ILC)) with the investigated S1-S3 glasses to show how the HVL of each differs^56^. Figure 17 shows that the currently available glasses outperform OC, HSC, and ILC materials as radiation shielding materials.

**Figure 18:**
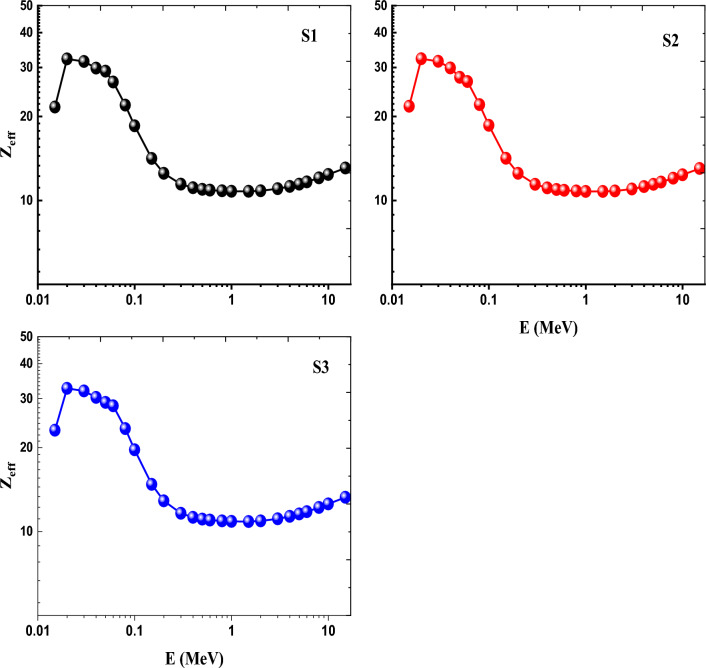
Variation of Z_eff_ as a function of photon energy of Ce (S1), Gd (S2) and Ce + Gd (S3) doped borosilicate glasses.

To explain the shielding characteristics of materials, the effective atomic numbers (Z_eff_) parameter is frequently utilized^13–17^. Figure 18 illustrates the difference of the (Z_eff_) as a function of photon energy (E) for all created rare earth glasses. The Z_eff_ trend indicates that adding rare earth oxides to modern glasses has a beneficial effect on raising Z_eff_ values by the order (S3)_Zeff_ > (S1)_Zeff_ > (S2)_Zeff_. This could be explained by the Z_eff_, which is highly reliant on the glass density. As a result, mixed Ce and Gd doped glass displayed the highest Z_eff_ factor.

## Conclusion

The three prepared CeO_2,_ Gd_2_O_3_ and CeO_2_ + Gd_2_O_3_ doped borosilicate glasses revealed precious optical, structural, physical and shielding characteristics towards the impact of ionizing gamma irradiation. The optical UV–Visible NIR absorption spectra revealed main UV peaks at 351 nm and 348 nm originated from Ce^3+^ and Gd^3+^ ions, respectively and assigned to 4f → 5d transition, while Ce + Gd doped glass depicted a peak at ~ 370 nm referring to the presence of Ce within the glass network rather than Gd. Irradiating glasses with 100 kGy of gamma rays displayed a minor reduction in the UV absorption bands and appearance of secondly peaks at 480, 430 and 500 nm for Ce, Gd and Ce + Gd doped glasses, respectively are correlated to the possible crystal field splitting of 5d (2p) states in Ce and Gd ions. Optical band gap energy (*E*_*g*_) displayed a slight reduction after 100 kGy of gamma irradiation, where $\text{\% }{E}_{g}$ is the smallest for Ce doped glass sample (20.2%) and the largest for the Gd glass sample (29.6) and in between for Ce + Gd doped glass sample (21%).

Photoluminescence spectra displayed asymmetric broad excitations identify the trivalent transitions of Ce^3+^ and Gd^3+^ ions due to 4f → 5d transitions, and emission intense bands at 412, 434, and 417 nm with a fixed extended emission band centered at 360 nm for CeO_2_, Gd_2_O_3_, and (CeO_2_ + Gd_2_O_3_) doped glasses, respectively. Emission spectrum of the mixed rare earth glass can be attributable to charge transfer bands, ^8^S_7/2_ → ^6^I transitions of Gd^3+^ ions, and 4f → 5d transitions of Ce^3+^ ions. Also, the intensity of photoluminescence spectra is slightly reduced by irradiation. CIE chromaticity coordinates are equally distributed and centered in the blue region showing that Gd^3+^ ions increase the luminescence of Ce^3+^ and more effectively transmit energy to Ce^3+^.

FTIR spectra of the glasses revealed a compacted network structures consist mainly of the tetrahedral building units of silicate (SiO_4_) and the trigonal (BO_3_) and tetrahedral (BO_4_) of borate. After irradiation with 100 kGy of gamma radiation the spectra revealed a large stability due to the rigid structure of the glassy network because of the pervious building units and presence of many TM ions e.g., Mn^2+^, Cr^3+^, Ti^4+^, and Fe^3+^ that fascinate radiation instigated-deficiencies centers by their adjustable outermost configurations. In addition to the doped rare earth ions; Ce^3+^ and Gd^3+^ that enhance the stability of glass structure owing to their large atomic numbers. ESR spectra revealed also the same spectra after irradiation referring to the absence of free radicals or breaking in bonds. Microhardness measurements showed also that CeO_2_ and/or Gd_2_O_3_ act as glass modifiers and increase the glass compactness with strong bond connections and higher hardness. Furthermore, thermal expansion coefficient (CTE), glass transformation temperature (T_g_), and dilatometric softening temperature revealed quite stability after irradiation especially the glass doped CeO_2_ and Gd_2_O_3,_ however adding CeO_2_ only causes a drop in thermal expansion coefficients and a rise Tg, and Td.

Such large stability in optical spectra, FTIR and ESR spectra indicates the highly compacted and rigid structures of the prepared glasses and their high tendency to avoid the effect of ionizing radiation. These performances approved by calculating radiation shielding parameters using the friendly online Phy_-_X/PSD program such as mass (MAC), linear attenuation coefficients (LAC), and Z_eff_ factor, revealing the order of (Ce + Gd) > (Ce) > (Gd)_,_ and the opposite order of half value layer (HVL), indicating high shielding efficiency of the glasses specially Ce + Gd doped glass. Comparing the prepared glasses with other commercial concrete materials designated also the high radiation ability of the investigated glasses.

The whole study exhibits the hopeful and economic usages of the useless MSW ashes to produce promising radiation shielding glasses with precious properties like high microhardness, high thermal and structural stability, interesting optical properties and valuable radiation shielding ability especially 0.5 CeO_2_ + 0.3 Gd_2_O_3_ doped borosilicate glasses that revealed the highest stability towards ionizing radiation and the greatest radiation shielding efficiency.

## Data Availability

The datasets used and/or analysed during the current study available from the corresponding author on reasonable request.
